# Mannich base limits *Candida albicans* virulence by inactivating Ras-cAMP-PKA pathway

**DOI:** 10.1038/s41598-018-32935-9

**Published:** 2018-10-08

**Authors:** Satish Kumar Rajasekharan, Chakkaravarthi Kamalanathan, Vinothkannan Ravichandran, Arvind Kumar Ray, Ann Susan Satish, Sucharitha Kannappan Mohanvel

**Affiliations:** 1 Centre for Research and Development, PRIST University, Thanjavur, 614-904 Tamil Nadu India; 2 Shandong University – Helmholtz Institute of Biotechnology, State Key Laboratory of Microbial Technology, School of Life Science, Shandong University – Qingdao campus, Aoshanwei, P. R. China; 3 ICAR-Central Institute of Brackishwater Aquaculture, Chennai, 600028 India; 4 Department of Biotechnology, Holy Cross College, Tiruchirappalli, 620-020 Tamil Nadu India; 5 Department of Biotechnology, D.G. Vaishnav College, Arumbakkam, Chennai, 600106 India

## Abstract

Mannich bases and its derivatives are regarded as supreme pharmacophores in therapeutics. The study investigates the antimycotic potential of Mannich bases, 1-((1H-benzimidazol-1-yl) methyl) urea (C1) and 1-((3-hydroxynapthalen-2-yl) methyl) thiourea (C2), against *Candida albicans*. Biofilm and hyphal inhibitory activities of the Mannich bases were tested by crystal violet quantification, fluorescence imaging cAMP rescue, qRT PCR, and by molecular docking analysis. The compounds inhibited the biofilms of *C. albicans* and restrained the filamentation abilities of the pathogen. Structure-activity relationship studies revealed that the presence of urea or thiourea moiety in the tail section is essential for interacting with adenylate cyclase (AC). The Mannich bases seemed to block Ras-cAMP-PKA pathway by inhibiting second messenger activity required for hyphal induction and biofilm formation. In conclusion, the study warrants point-of-care testing of C1/C2 and provides a starting point for deriving several structurally modified Mannich bases which might plausibly replace the prevailing antimycotic drugs in future.

## Introduction

*Candida albicans* is the most prevalent pathogenic yeast known to cause a diverse spectrum of systemic and chronic infections in human^1^. In the last few decades, *C. albicans* infections have resulted in high mortality rate ranging from 15 to 25% of the world population^2^. Biofilm formation and hyphal transition are the major virulence traits in *C. albicans* which remain as the vital etiological factors of candidiasis^3^. This ability allows the pathogen to adhere to host cells, invade into intestinal epithelium and colonize to form sessile phenotypes^4^. Yeasts constituting biofilm populations are reported to have an unique characteristic feature of exhibiting resistance to antimicrobials and antibiotics^5^. Extracellular and polymeric matrix structures of biofilms give cohesive and adherent properties of the organism to a surface which render the species more resistant to antifungal agents^5^. Several approaches were taken into consideration to eradicate *C. albicans* biofilms^6,7^.

In recent years, several mycologists have reported the rapid emergence of drug-resistant strains in the clinical specimens^8^. Present treatment strategies are crippled due to inadequate antimycotic drugs and the rapid emergence of drug-resistant variants (Li *et al*., 2017). Alternatively, targeting Ras-cAMP pathway is an alternate and selective approach to counter the virulence of *C. albicans*^9^. The obvious role of Ras1 as a switching G-protein in *C. albicans* is established^10^. Inhibitors of Ras pathway selectively target the signals and/or enzymes required for proper functioning of cellular metabolism^11^. Adenylyl cyclase, Cyr1, is an enzyme which is shown to regulate several developmental and virulence factors in *C. albicans*^12,13^. These include hyphal filamentations, white-opaque phenotypic switching, and biofilm formations^13^. The enzyme converts ATP to cAMP and the resultant cAMP binds to and activates the catalytic subunit (Bcy1) of cAMP responsive protein kinase (PKA)^14,15^. PKA, in turn, phosphorylates the transcription factor, *EFG1*, which triggers biofilm formations and filamentations in pathogenic yeasts^14,16^. Lately, few studies have reported inactivation of AC by different phytocompounds^17^ and several studies have shown the expressional levels of Ras-cAMP related genes in *C. albicans*, and theorized the involvement of this signaling pathway in inducing the anti-hyphal effect.

Mannich bases are end products of a nucleophilic addition reaction termed as a Mannich reaction, and are often referred to as beta-amino ketones. These synthetic chemicals have played a vital role in the development of medicinal and pharmaceutical chemistry as well. They often act as chemical leads for the synthesis of new pharmacophores or drugs with medicinal values. Several clinically reliable Mannich bases have been reported in recent times^18^. Other studies have reported the antifungal, antimycobacterial, antimalarial, and/or antiviral potential of Mannich bases and their derivatives^19–21^. Due to the emerging antibiotic resistance in microbial species have evolved, there is a constant demand to develop innovative, effective, and affordable drugs to fight the existing antibiotic crisis. This study was conducted to test the antibiofilm and antihyphal potency of few synthetic Mannich bases on *C. albicans* strains. A urea/thiourea-tail containing Mannich base was found to inhibit the biofilms and hyphal elements of *C. albicans*. In-depth analysis was performed to identify and elucidate the mode of action of the lead compound by molecular docking, quantitative PCR, and cAMP rescue assays.

## Materials and Methods

### Ethics statement

All the experiments were approved by the Ethical Committee of PRIST University, Thanjavur, India and the methods were carried out as per the guidelines.

### Mannich bases, strains and culture conditions

**Figure 1:**
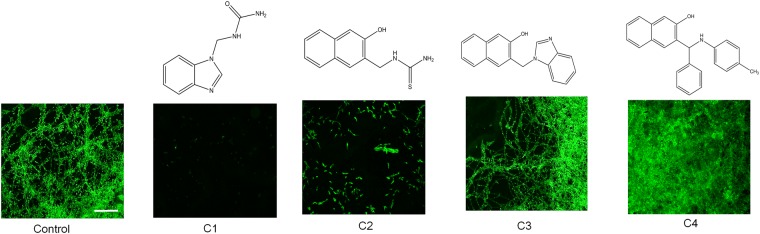
Structures and antibiofilm activities of Mannich bases tested against *C. albicans*. The non treated controls showed dense biofilms and hyphal morphology on glass surfaces when viewed under confocal microscope after 48 h of incubation at 37 °C and subsequent staining with 0.1% acridine orange. Biofilm inhibition was observed in 1-((1H-benzimidazol-1-yl) methyl) urea (C1), and 1-((3-hydroxynapthalen-2-yl) methyl) thiourea (C2), while compounds, 3-((1H-Imidazol-1-yl)methylnaphthalene-2-ol (C3), and 3-((1 H-Benimidazol-1-yl) methyl) napthalen-2-ol (C4) did not inhibit yeast biofilms. Scale bar: 50 µm.

Mannich bases, [1-((1H-benzimidazol-1-yl) methyl) urea (C1),1-((3-hydroxynapthalen-2-yl)methyl) thiourea (C2)3-((1H-Imidazol-1-yl)methylnaphthalene-2-ol (C3), and 3-((1 H-Benimidazol-1-yl) methyl) napthalen-2-ol (C4) reported in the study were synthesized by procedures as previously described^22^. The structures of all the Mannich bases are presented in Fig. 1. *C. albicans* strains (ATCC 90028 (MTCC 3017), MTCC 183, and MTCC 227) used in the experiments were procured from the American Type Culture Collection (ATCC) or Microbial Type Culture Collection (MTCC, India). All strains of *C. albicans* were stored in the culture medium (yeast extract- peptone- dextrose, YPD) as frozen stock with 10% (v/v) of glycerol at −80 °C and sub-cultured twice at 37 °C before every single usage of yeast culture.

### Minimum inhibitory concentration assay

Microdilution assays were done to determine minimum inhibitory concentrations (MIC) in a 96-well polystyrene plates (Tarson, India)^23^. *C. albicans* were cultured in spider medium (1% mannitol, 1% nutrient broth, 0.2% K_2_HPO_4_, pH 7.2) with 0.165 M morpholine propane sulfonic acid (MOPS) buffer in 96-well flat-bottomed microtitre plates followed by inoculation of different Mannich bases (0, 8, 16, 32, 64, 128 and 256 µg/mL). After incubation at 37 °C for 48 h, MIC was determined by measuring the optical density at 600 nm and interpreting the lowest concentration of Mannich base that inhibits the microbial growth by at least 80% and comparing the readings with organisms grown without Mannich bases. The experiments were performed in triplicates and the background optical densities were subtracted from each well of the 96-well polystyrene plates.

### *In vitro* Biofilm inhibition assay

Biofilm inhibition assays were performed in 96-well polystyrene plate (Tarsons, India)^5^. *C. albicans* overnight-grown culture of 1 × 10^6^ cells/mL were prepared in spider medium and were inoculated with Mannich bases at different concentrations (0, 8, 16, 32 and 64 µg/mL). They were used as anti-biofilm agents while the wells without Mannich base served as controls. After incubation for 48 h at 37 °C, followed by removal of non-adhered cells with sterile PBS, the biofilms were quantified by staining with 0.4% crystal violet for 20 mins. The crystal violet was rinsed thrice with H_2_O and extracted with 95% ethanol. The absorbance was measured by Bio-Tek Synergy 4 microplate reader (Thermo Fischer, USA) at 575 nm. The experiments were performed in triplicates and results are presented as mean ± SEM. cAMP biofilm rescue assays are expressed in percentage biofilm inhibition. The percent biofilm inhibition was calculated by the below formula:

$${\rm{Percent}}\,{\rm{biofilm}}\,{\rm{inhibition}}=[({\rm{Control}}\,{{\rm{OD}}}_{575{\rm{nm}}}-{\rm{Test}}\,{{\rm{OD}}}_{575{\rm{nm}}})/{\rm{Control}}\,{{\rm{OD}}}_{575{\rm{nm}}}]\times 100$$

### Microscopic imaging

For imaging, *C. albicans* strains (1 × 10^5^ cells/mL) were cultured in spider medium grown on 96-well polystyrene surfaces (Tarsons, India) and incubated at 37 °C for 48 h with or without Mannich bases. After incubation, the non-adherent cells were removed by washing with sterile PBS thrice followed by aspirating the supernatant. The sessile cells adhered firmly on the bottom of the plates and were stained with 0.4% crystal violet and/or 0.1% acridine orange. The images were visualized and captured under a fluorescent microscope (Nikon Eclipse Ti 100, Japan) and documented.

### XTT reduction assay

Biofilm quantification was performed by calorimetric XTT [2,3-bis (2-methoxy-4-Nitro-5-sulfophenyl)-2H-tetrazolium-5-carboxanilide sodium salt] reduction assay and viability was indicated in terms of the calculated metabolic activity percentage. Experimental procedures involve inoculation of *C. albicans* strain in spider medium for 48 h at 37 °C with shaking at 250 rpm, and proceeding to re-inoculate the cells in spider medium with or without Mannich base (0, 8, 16, 32 and 64 µg/ml) in a 96-well plate for 24 h at 37 °C. XTT reduction assay kit (Sigma–Aldrich, USA) was used to study biofilm metabolic activities and experiments were performed as described by the manufacturer. The colored supernatant was measured at 450 nm using a Bio-Tek Synergy 4 microplate reader (Thermo Fischer, USA).

### Yeast-Hyphae (Y-H) inhibition assay

The yeast cell suspension was incubated in liquid spider medium with different concentrations of Mannich base (0, 8, 16, 32 and 64 µg/ml) at 37 °C with continuous shaking (250 rpm) for 24 h. Bright-field and fluorescent microscope were used to visualize the formation of true hyphae in the aliquots. The aliquots were smeared, fixed, and stained with 0.4% crystal violet (CV) or 0.1% acridine orange. Inhibition of hyphal protrusion from embedded colonies was tested by streaking the yeast cells in spider medium and by visualizing the colonies after 5 days of incubation under dark field optical imaging (Nikon Eclipse Ti 100, Japan).

### Quantitative Real-Time PCR assays

Quantitative RT-PCR was performed by protocols as previously described^23^. Planktonic cells of *C. albicans* treated with C1 (32 µg/mL) and grown in spider medium at 37 °C for 24 h are diluted to the cell density of 1.0 × 10^6^. They were harvested by centrifugation at 10,000 × g for 10 min. Approximately 1 µg of total RNA was used to synthesize cDNA using random primers using cDNA synthesis kit (TAKARA, USA) RT- PCR was performed in 8-tube strips using 2X USB® VeriQuest TM Fast SYBR® Green qPCR master mix (Affymetrix, Inc. USA) in a Real-Time PCR machine (Step One system, Applied Biosystems, USA), and delta-delta Ct method was used to compute gene expression. Beta-actin was used as an internal reference gene and the primers used are listed in Supplementary Table 1^24^.

### Molecular docking analysis

Molecular docking study was executed by Schrodinger version 9.3. The ligands were flexible to rotate within the binding poses, while receptor was kept rigid. A grid was generated with close proximity to active sites of adenylate cyclase and docking was performed by Glide (XP model). The grid maps representing the center of active site pocket for the ligand were calculated with Autogrid. Glide module of Schrodinger 9.3 was used for docking Mannich bases with the crystal structure of adenylate cyclase (Code IFX2) retrieved from the Protein Data Bank (PDB) (10.2210/pdb1fx2/pdb) as described by Singh *et al*.^23,25^. Finally, the results generated were visualized by PyMOL viewer for analysis of minimum binding energy (Kcal/mol), Ki (Inhibition constant) value (μM), and hydrogen and hydrophobic interaction of the docked inhibitor to the modeled structure.

### cAMP Rescue experiment

cAMP rescue experiment was performed by the procedure as described previously^24^. Overnight-grown *C. albicans* cells were diluted in spider medium. Di-butyryl-cAMP (db-cAMP) (Santa Cruz Biotechnology, USA) were added to the culture with a final concentration of 5 mM immediately after treatments with C1 (32 µg/mL) and incubated for a period of 5 h, following which the yeast cells were assessed for hyphal growth. The free- drug treatment cells with or without db-cAMP served as a control. After incubation, cells were visualized under the phase contrast microscope. Inhibition of hyphal protrusion from embedded colonies was tested by streaking the yeast cells in spider medium and by visualizing the colonies after 5 days of incubation under dark field optical imaging (Nikon Eclipse 100, Japan).

### Statistical analysis

All experiments were conducted in triplicates, and the results were expressed as means ± SD. Student’s *t* test was used to determine the significance levels and was considered as statistically significant when *p* < 0.05 (*p < 0.05, **p < 0.01, and ***p < 0.001).

## Results and Discussion

Emergence of a multi-drug-resistant variant of yeast species via vertical and horizontal gene transfers demand the need for developing a new class of antifungals superior to the antibiotics used in the current regimen. The therapeutic importance Mannich bases are well documented^21^. This study identifies few newly synthesized Mannich bases as an antimycotic drug against the opportunistic pathogen, *C. albicans*. Initially, *in vitro* assays to test the effect of Mannich bases on *C. albicans* pathogenesis were conducted. *C. albicans* strain (ATCC 90028/ MTCC 3017) was initially used for screening the anti-pathogenic potential of Mannich bases due to its remarkable biofilm forming ability. Among the tested chemicals, C1 and C2 were efficient in reducing the yeast biofilms at the tested dosage (100 μg/mL), while C3 and C4 were ineffective (Fig. 1). Crystal violet quantification of yeast biofilms treated with Mannich bases and the structures are presented in Supplementary Fig. 1.

**Figure 2:**
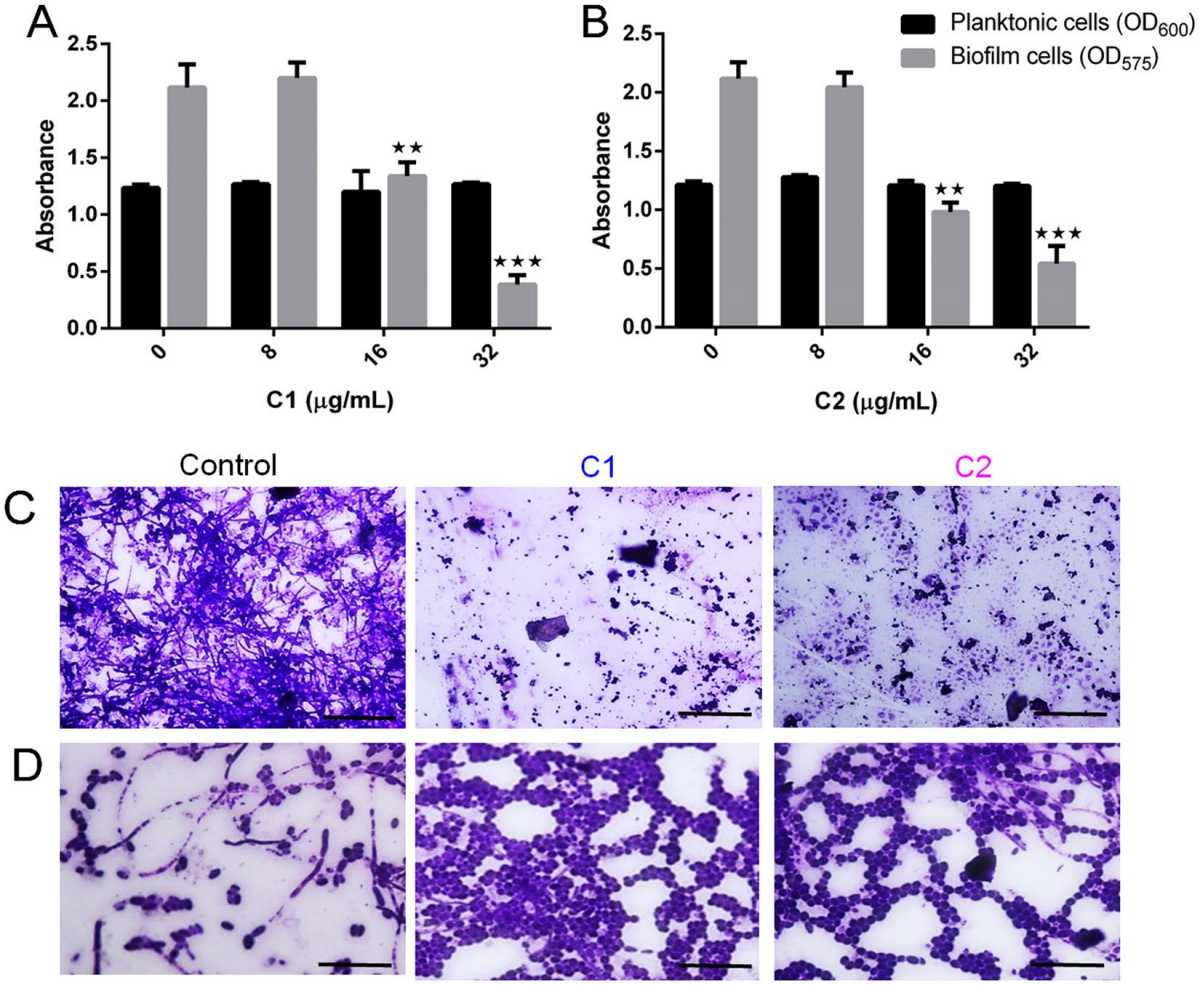
Effect of Mannich bases on *C. albicans* biofilms. (**A**) Inhibitory effect of 1-((1H-benzimidazol-1-yl) methyl) urea (C1) on *C. albicans*, C1 showed dose dependent inhibition of yeast biofilms after 48 h of incubation at 37 °C, with maximum inhibition at 32 µg/mL. (**B**) Inhibitory effect of C2 on *C. albicans*, 1-((3-hydroxynapthalen-2-yl) methyl) thiourea (C2) showed dose dependent inhibition of yeast biofilms after 48 h of incubation at 37 °C, with maximum inhibition at 32 µg/mL. (**C**) Light microscopic images of *C. albicans*, biofilms stained with 0.4% crystal violet showing maximum inhibition in C1 and C2 treated groups (Scale bar: 50 µm). (**D**) Effect of Mannich bases on yeast-to-hyphal transition in liquid spider media, control group show more hyphal elements per field following staining with crystal violet (0.4%) stain, treatment with C1 and C2 shows more yeast cells per field (Scale bar: 100 µm).

Based on the findings, further experiments were conducted with C1 and C2. *C. albicans* biofilm formation was tested in the presence and absence of the Mannich bases (0–32 μg/mL) using microtitre plates. At lower concentrations (16 and 32 μg/mL), C1 and C2 significantly inhibited the biofilm cells of *C. albicans* without compromising the planktonic counterparts (Fig. 2A,B). Microscopic inspection of 48 h grown biofilms on glass surfaces stained with 0.4% crystal violet (Fig. 2C) revealed a drastic reduction in biofilm cells in treated groups. The Mannich bases were also effective in preventing yeast-hypha transitions in liquid spider medium. Microscopic images of C1 or C2 treated *C. albicans* showed the presence of yeast cells and no filaments when cultured in liquid spider media (Fig. 2D). The aliquots were smeared, fixed and stained with Gram’s crystal violet (0.4%) and fluorescent stain (acridine orange (0.1%)) to visualize the yeast cells and the hyphal elements. (Fig. 2D). In C1 and C2 treated groups the filaments were absent and there were more yeasts per field, while the controls revealed more filaments per field.

**Figure 3:**
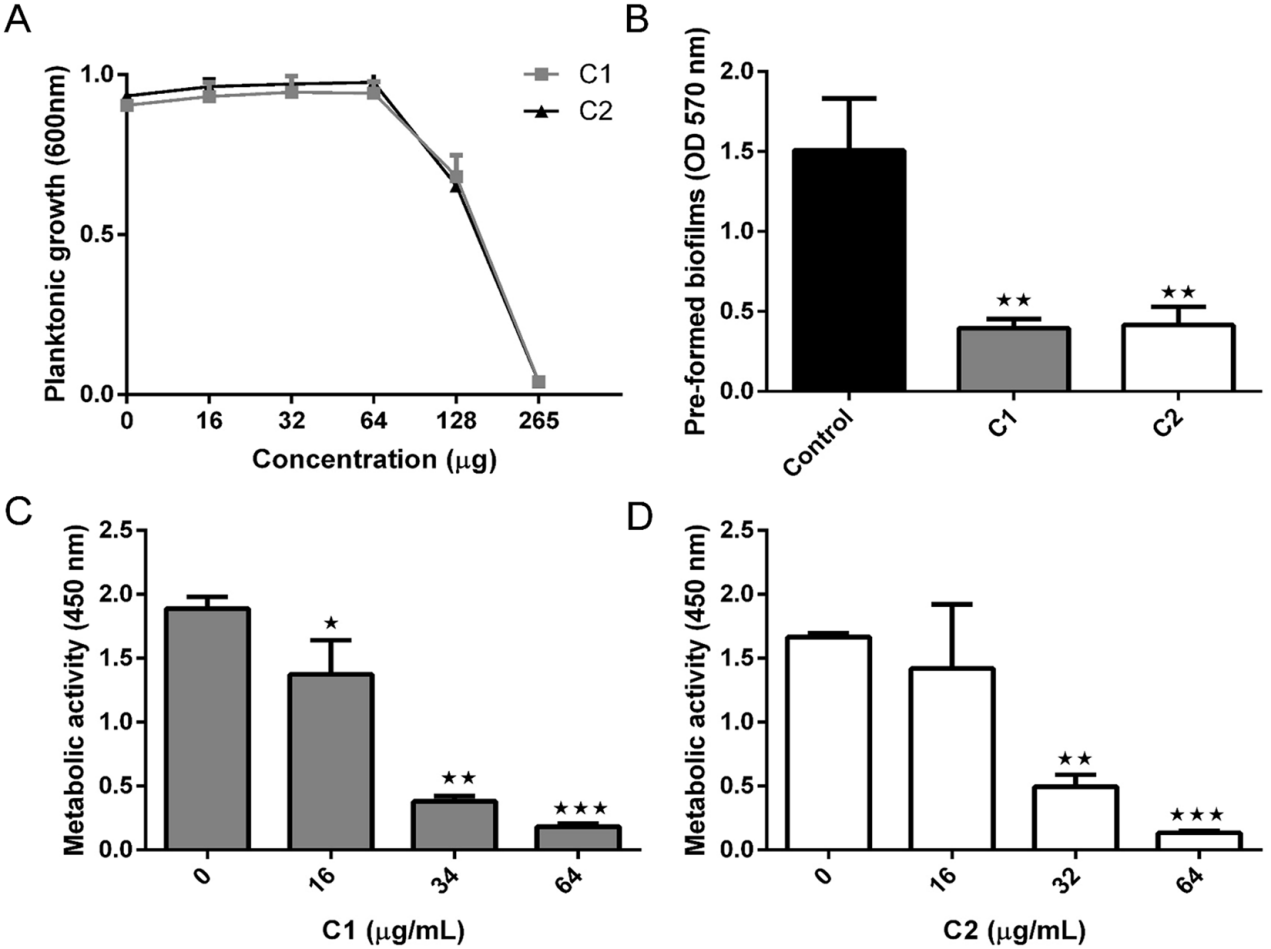
Effect of Mannich bases on growth, pre-formed biofilms and metabolic activities of *C. albicans*. (**A**) Concentration dependent decline of yeast growth when treated with 1-((1H-benzimidazol-1-yl) methyl) urea (C1) and 1-((3-hydroxynapthalen-2-yl) methyl) thiourea (C2), showing MIC_90_ as 256 µg/mL, (**B**) disruption of *C. albicans* biofilms in presence of C1 and C2. The compounds showed significant reduction in biofilms at 64 µg/mL, (**C**) metabolic activity of *C. albicans* treated with C1 showing a concentration dependent decline in the biofilm cells with a maximum inhibition at 64 µg/mL, and (**D**) metabolic activity of *C. albicans* treated with C2 showing a concentration dependent decline in the biofilm cells with maximum inhibition at 64 µg/mL. All the cultures were incubated at 37 °C for 48 h. Bar graphs represent means ± SD of n = 3 experiments. *P < 0.05, **P < 0.01, and ***P < 0.001 vs. the control.

Further, to confirm the efficacy, the compounds (32 μg/mL) were tested against several other *C. albicans* strains and astonishingly, C1 and C2 excelled in limiting the biofilms in the tested strains (Supplementary Fig. 2). MIC of C1 and C2 against the strain was estimated as 256 μg/mL (Fig. 3A). The compounds were also successful in disrupting the pre-formed *C. albicans* at 64 μg/mL, suggesting its therapeutic role (Fig. 3B). Importantly, activity of the compounds were further confirmed by XTT assay. XTT assay was performed which uses cell viability as a marker based on metabolic activity of the cells. The assay is based on the reduction of XTT (yellow coloured compound) to a bright orange color formazan derivatives by viable cells. C1 (Fig. 3C) and C2 (Fig. 3D) caused a dose-dependent decline in the metabolic activities of *C. albicans* biofilm cells, thus confirming the crystal violet quantification methods.

**Figure 4:**
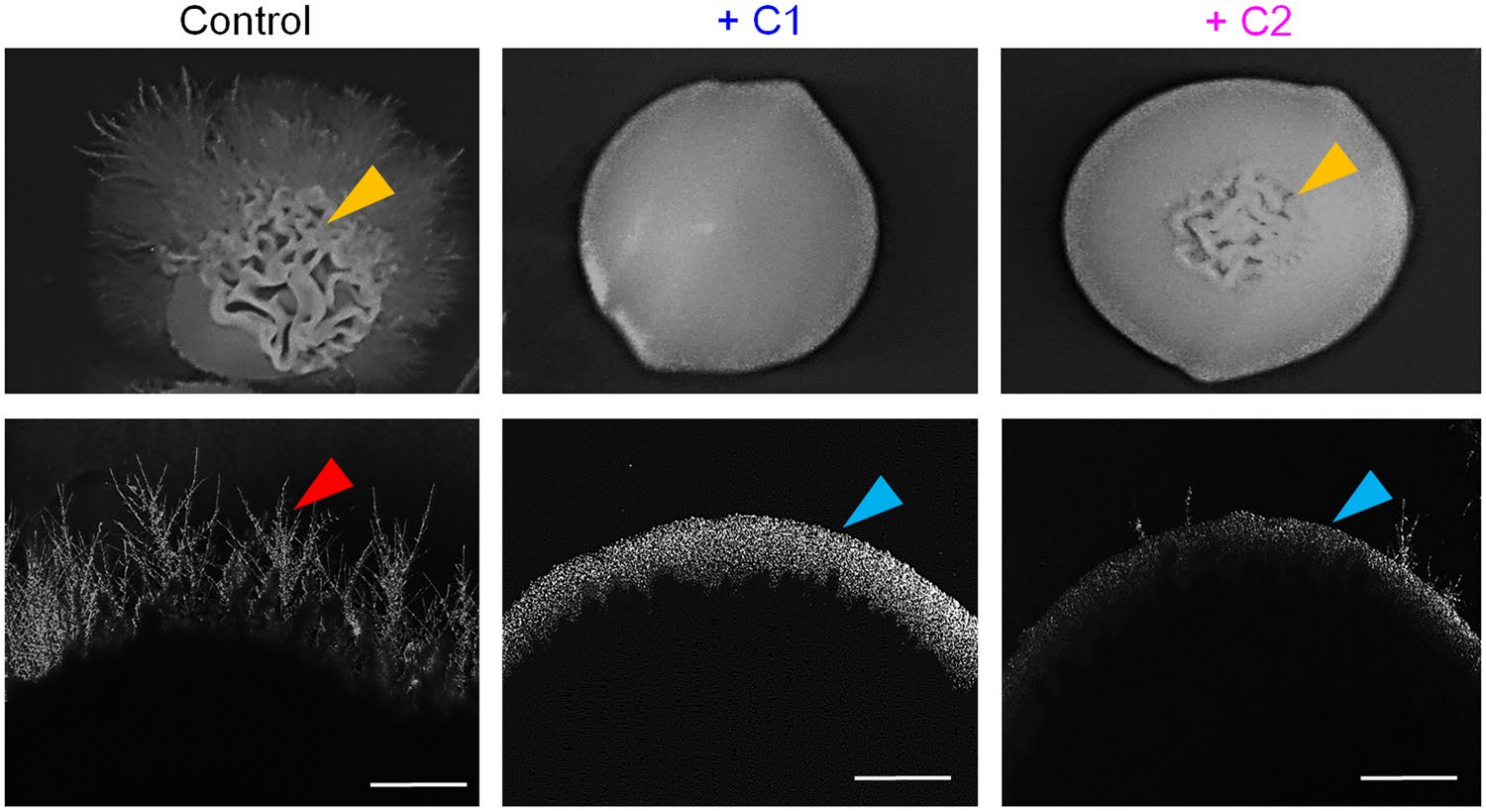
Effect of Mannich bases on yeast-to-hyphal transition on solid media. The control groups show extensive hyphal protrusion from embedded colonies and top filamentation that are absent in 1-((1H-benzimidazol-1-yl) methyl) urea (C1) treated colony and comparatively low in 1-((3-hydroxynapthalen-2-yl) methyl) thiourea (C2) treated colony. The colonies were imaged after five days of incubation at 37 °C. Yellow arrow denotes top filamentations observed in control and C2 treated colony, red arrow shows hyphal protrusion from colony edges, and blue arrow represents smooth topography. Scale bar: 10 µm.

*C. albicans* colonies on solid spider medium also revealed extensive hyphal protrusion, irregular shape, and top filamentation after five days of incubation at 37 °C (Fig. 4). C1 (32 μg/mL) treated colonies were devoid of hyphal protrusions and revealed smooth surface topography as confirmed by colony morphology and dark-field microscopic imaging of colony edges (Fig. 4). C1 also was devoid of top filamentations and exhibited round morphology. C2 was also successful in limiting the hyphal protrusion from colony edges and exhibited smooth and round morphology, though revealed low levels of top filamentations. Overall, hyphal inhibition in both liquid and solid media affirm C1 and C2 as strong hyphal inhibitors.

Hyphal and biofilm formation *in C. albicans* requires activation of the transcriptional regulator Nrg1p, which functions as a negative regulator of Y-H morphogenetic transitions. Activation of *NRG1* gene follows two major pathways namely Ras-dependent (Ras-cAMP-PKA) and Ras-independent (Ubr1-Cup9) pathway^26^. Ras-dependent or cAMP-PKA pathway functions by producing cAMP required for activation of protein kinases (PKA) via AC. AC is a candidate enzyme which plays a vibrant role in modulating Ras-cAMP-PKA pathway and effectively controls filamentation traits in *C. albicans*^11^. Molecular docking was performed to understand the interactions of the Mannich bases with the ATP binding pocket of AC enzyme. Glide (grid-based ligand docking energetic) module of Schrodinger 9.3 was used for docking the ligands (C1-C4, quercetin and 2′, 5′-Dideoxyadenosine 3′-triphosphate tetrasodium salt (standard)) with the crystal structure of AC^23,25^. Quercetin was taken as the positive control as it was previously shown to limit hyphal filamentations in *C. albicans* by interacting with AC^25^. Among the tested ligands, C1 exhibited the best negative energy Glide scores (−5.90 kcal/moL) followed by C2 (−4.62 kcal/moL) (Table 1). The compounds, mainly C1, showed better binding efficiencies when compared to the standard (−4.86 Kcal/moL), and quercetin (−4.92 Kcal/moL). Glide scores of the tested ligands are presented in Supplementary Fig. 3. The other Mannich bases (C3 and C4) showed lesser binding energy scores which might be attributed to their complex structures (Table 1).

**Table 1:** Interactions of Mannich bases and standard inhibitors with adenylate cyclase.

S.No	Mannich bases	GScore (kcal/moL)	H- Bond (s)	Key residue (s)
1	1-((1H-benzimidazol-1-yl) methyl) urea (C1)	−5.90	2	Asp^83^, Asn^1017^
2	1-((3-hydroxynapthalen-2-yl)methyl) thiourea (C2)	−4.62	2	Thr^979^, Asn^1017^
3	3-((1H-Imidazol-1-yl)methylnaphthalene-2-ol (C3)	−2.92	1	Ser^1013^
4	3-((1 H-Benimidazol-1-yl) methyl) napthalen-2-ol (C4)	−3.065	1	Asn^1017^
5	Quercetin	−4.92	2	Leu^973^, Asn^980^
6	2′,5′-Dideoxyadenosine 3′-triphosphate tetrasodium salt (Standard)	−4.86	2	Ser^1013^. Asn^980^

**Figure 5:**
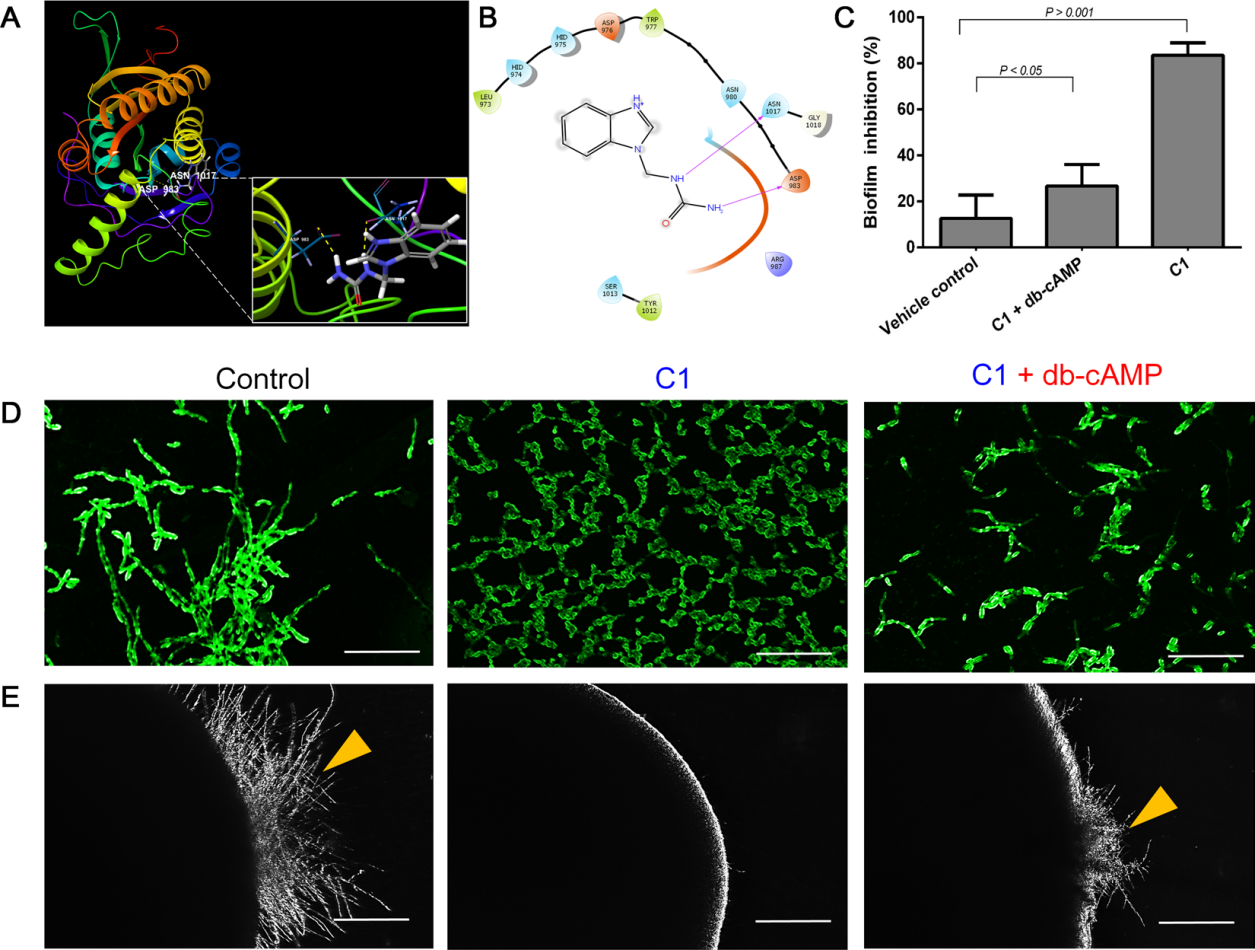
Interaction of Mannich bases with adenylate cyclase and hyphal rescue using exogenous db-cAMP. (**A**) 3D representations of interactions of 1-((1H-benzimidazol-1-yl) methyl) urea (C1) with adenylate cyclase (AC), (**B**) 2D representation of C1- adenylate cyclase complex. (**C**) Percent *C. albicans* biofilm inhibition was calculated, which showed maximum inhibition percentage for C1 (~83%) treated cells (*P* < 0.001 vs. the control), while the inhibitions were ~12% (*P* > 0.05 vs. the control) and ~26% (*P* > 0.05 vs. the control) for db-cAMP (vehicle control), and C1 + db-cAMP, respectively, (**D**) hyphal conformations of *C. albicans* on liquid spider media after fixing and staining with 0.1% acridine orange. Control groups show more hyphal elements per field, treatment with C1 shows more yeast cells per field, while C1 (32 µg/mL) + db–cAMP (5 mM) shows more pseudo-hyphal elements suggesting db–cAMP mimics cAMP and triggers the Ras-cAMP pathway and (**E**) hyphal protrusion of *C. albicans* colonies embedded in solid spider medium observed on fifth day of streaking and incubation at 37 °C, treatment with C1 (32 µg/mL) shows smooth colony edges, while C1 (32 µg/mL) + db–cAMP (5 mM) shows the reoccurrence of hyphal filaments from embedded colonies. Yellow arrow denotes top filamentations observed in control and C1 + db–cAMP treated colony. Scale bar: 100 µm.

Interactions of C1 with ATP-binding pocket of adenylate cyclase revealed strong binding interactions with two backbone hydrogen bonds with catalytic amino acids namely, asparagine-1017 and aspartic acid-983 (Fig. 5A,B), while C2 formed H-bonds with threonine-979 and asparagine-1017 (Table 1). The interactions were further stabilized by Van der Waals force interaction forces with polar and non-polar amino acid residues within the active site. Structure-activity relationship studies of C1-AC and C2-AC complexes revealed that the presence of urea or thiourea moiety in the tail section is vital for H-bonding interactions (Fig. 1). Previously, it was shown that a potent antibacterial compound containing a urea moiety, aminobenzimidazole urea, inhibited bacterial gyrase (GyrB) and topoisomerase IV (ParE)^27^. Taken together, the docking results confirm the hyphal inhibition by C1 and C2 and the results were in agreement.

To confirm that the observed anti-biofilm and anti-hyphal effect might be due to AC inactivation, we performed cAMP rescue experiment to validate our findings^17^. *C. albicans* biofilm inhibition was tested in the presence of C1 (32 µg/mL), C1 (32 µg/mL) + db-cAMP (5 mM), and db-cAMP (5 mM) alone as vehicle, and subsequently stained with crystal violet (0.4%) and quantified at 575 nm using a microtitre plate reader. Percent biofilm inhibition was calculated which showed maximum biofilm inhibition percentage for C1 (~83%) treated cells, while the inhibitions were ~12% and ~26% for db-cAMP (vehicle control), and C1 + db-cAMP, respectively (Fig. 1C). The data confirms the role of cAMP in biofilm formation. For hyphal assay, overnight cultures of *C. albicans* in liquid spider medium were supplemented with dibutyryl-cAMP (db-cAMP) (Santa Cruz Biotechnology, USA) (Final concentration: 5 mM) immediately after treatment with C1 (32 µg/mL) and incubated for a period of 5 h, following which the yeast cells were assessed for hyphal growth. Reassuringly, the exogenous supply of db-cAMP to C1 treated *C. albicans* partially restored the hyphal phenotypes (Fig. 5D). Cell counts showed a preponderance of yeast cells in C1 treated groups (>90%), while the groups treated with C1 and db-cAMP showed decreased yeast cells (20%) and dominance of pseudohyphal elements (80%). Further, assays on solid spider medium supplemented with and without C1 (32 µg/mL), and C1 (32 µg/mL) + db-cAMP (5 mM) also revealed fewer hyphal protrusion on from colony edges in C1 + db-cAMP treated groups (Fig. 5E). Explaining this finding, we suggest that db-cAMP mimics cAMP, binds to the protein kinases (PKA), and activates *EFG1*, which in turn triggers biofilm and hyphal formations.

**Figure 6:**
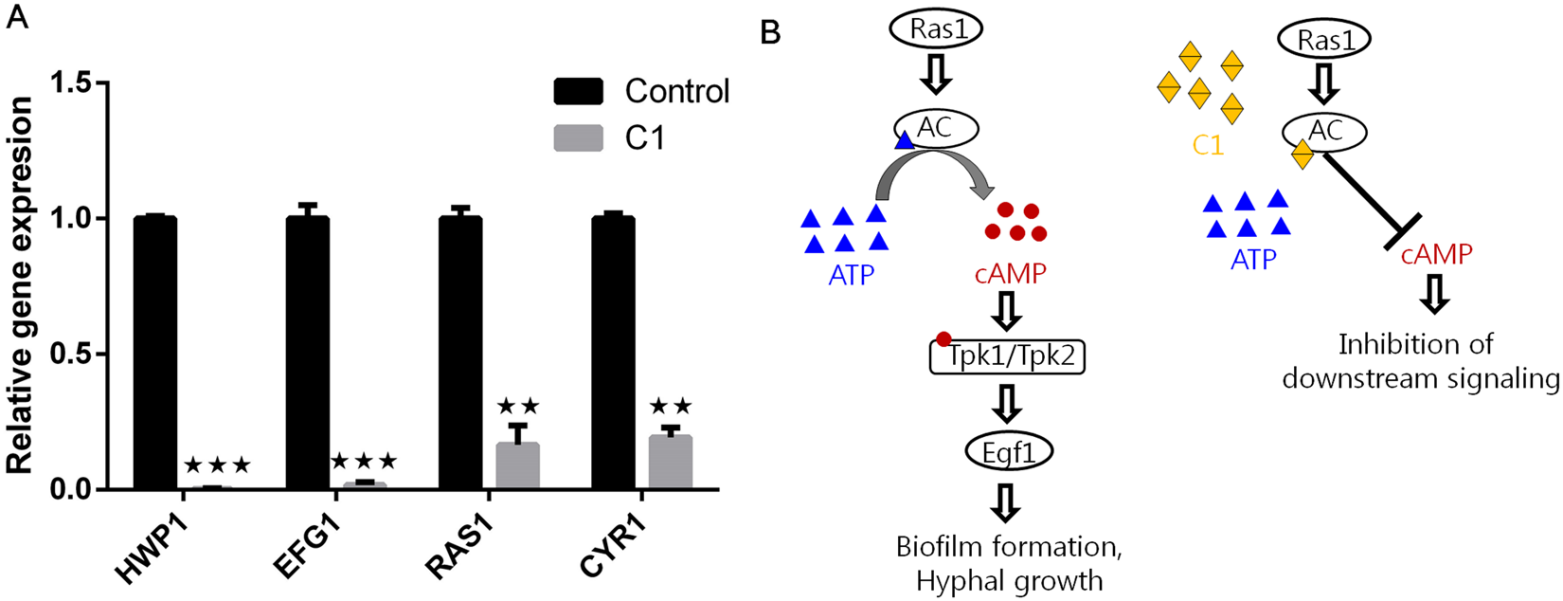
(**A**) mRNA transcript levels of candidate genes involved in Ras-cAMP pathway in the presence and absence of 1-((1H-benzimidazol-1-yl) methyl) urea (C1), the results show significant down-regulation of mRNA transcripts (*RAS1* by >6 fold, *CYR1* by >5 fold, by *EFG1* >56 fold and *HWP1* by >500 fold respectively) in C1 treated cells, the quantification was performed by 2^−ΔΔCT^ using ß-actin as internal reference gene, (**B**) plausible mode of action of C1 suggesting its potential involvement in binding to ATP binding sites of adenylate cyclase, thus inhibiting the production of cAMP levels required for biofilm and hyphal growth. Bar graph represents means ± SD of n = 3 experiments. *P < 0.05, **P < 0.01, and ***P < 0.001 vs. the control.

C1 exhibited the best activity against *C. albicans* biofilms, hyphal elements and showed remarkable interactions with AC. Hence the compound was further taken forward to understand the mechanism involved in hyphal inhibition, we performed quantitative PCR analysis of candidate genes involved in Ras- Ras-cAMP-PKA pathway (*RAS1* (Ras GTPase), *EFG1* (enhanced filamentation growth factor-1), *CYR1* (adenylate cyclase)), and the hyphal concomitant gene, *HWP1* (hyphal wall protein-1). The results revealed significant down-regulation (*RAS1* by >6 fold, *CYR1* by >5 fold, by *EFG1* >56 fold and *HWP1* by >500 fold respectively) in C1 (32 µg/mL) treated groups, thus confirming the involvement of Ras-cAMP pathway in the observed anti-hyphal effect (Fig. 6A). *EFG1* is a key regulator which function downstream Ras1 and is known to control biofilm formation and hyphal morphogenesis^28^. Significant downregulation of downstream-activating genes (*EFG1* and *HWP1*) suggests an upstream inhibition in the Ras-cAMP-PKA signaling pathway. Ras1 controls the expression of cyr1, and downregulation of *CYR1* and *RAS1* mRNA transcripts observed in our experiment (Fig. 6A). It also suggests that C1 might block Ras1 as well, although we were unable to clarify this finding.

Ras-cAMP-PKA pathway is unanimous in all eukaryotes and is essential for cellular response and metabolism^9,29^. Ras plays a critical role in controlling several phenotypes in *C. albicans* which includes yeast/hyphal morphogenesis, finger and tentacle formations, cell adhesion and biofilm formation, and MTLa/an opaque cell morphology. When activated, Ras, a GTPase protein interacts with AC in the plasma membrane and initiates the conversion of ATP to cAMP. cAMP binds and activates PKAs (TPK1/TPK2) which further initiates a signaling cascade leading to cellular response, hyphal growth and biofilm formation in yeasts^9^. Inactivation of AC, in turn, lowers cAMP levels thus inactivating PKA and enzymes downstream Ras-cAMP-PKA pathway^30^. From our findings, we suggest that the Mannich bases might function as competitive inhibitors and achieve hyphal and biofilm inhibition by inactivating AC (Fig. 6B).

The use of an antibiofilm agent for antimicrobial chemotherapy is an effective measure to control the microbial disease. Several reports have established the use of synthetic compounds as a promising biofilm inhibitor^31^. In this present study, we tested the antibiofilm and anti-hyphal potential of two synthetic Mannich bases and this is the first report to the best of our knowledge. We also propose the mode of action of the Mannich bases and have explained the possible role of urea moiety in inducing the anti-pathogenic effect. Our pivot warrants point-of-care testing of the compounds and provides a developmental starting point for deriving several structurally modified Mannich bases which might plausibly replace the existing antimycotic drugs to treat candidiasis in near future.

## Electronic supplementary material

Supplementary Information
