# Supplementary material for: Mannich base limits Candida albicans virulence by inactivating Ras-cAMP-PKA pathway
Source: Sci Rep. 2018 Oct 8;8:14972. doi: 10.1038/s41598-018-32935-9 (PMC6175908; doi:10.1038/s41598-018-32935-9)
Supplement: Supplementary file 1 — Supplementary Information [file 41598_2018_32935_MOESM1_ESM.docx]

**Mannich base limits *Candida albicans* virulence by inactivating Ras-cAMP-PKA pathway**

Satish Kumar Rajasekharan^1†*^, Chakkaravarthi Kamalanathan^1†^, Vinothkannan Ravichandran^2^, Arvind Kumar Ray^3^, Ann Susan Satish^4^, and Sucharitha Kannapan Mohanvel^5*^

^1^Centre for Research and Development, PRIST University, Thanjavur 614-904, Tamil Nadu, India.

^2^Shandong University – Helmholtz Institute of Biotechnology, State Key Laboratory of Microbial Technology, School of Life Science, Shandong University – Qingdao campus, Aoshanwei, P. R. China.

^3^Central Institute of Brackishwater Aquaculture-ICAR, Chennai - 600028, India

^4^Department of Biotechnology, Holy Cross College, Tiruchirappalli 620-020, Tamil Nadu, India

^5^Department of Biotechnology, D.G. Vaishnav College, Arumbakkam, Chennai-600106 –India

**Running title**: Mannich bases limits *Candida* virulence

^†^**Equal contributions**

**^*^Corresponding authors:** [generic.sat@gmail.com](mailto:generic.sat@gmail.com) and [sucharithamohanvel@gmail.com](mailto:sucharithamohanvel@gmail.com).

| **Genes** | **Function** | **Sequence (5’ - 3’)** | **Amplicon (bp)** |
| --- | --- | --- | --- |
| *RAS1* | GTpase | Sense : GAGGTGGTGGTGTTGGTA  Antisense: TTCTTCTTGTCCAGCAGTATC | 161 |
| *EFG1* | Transcription regulator | Sense : ATTGAGATGTTGCGGCAGGATAC  Antisense: ACTGGACAGACAGCAGGAC | 99 |
| *CYR1* | Adenylate cyclase | Sense : GTTTCCCCCACCACTCA  Antisense: TTGCGGTAATGACACAACAGA | 114 |
| *HWP1* | Hyphal wall protein | Sense : ACAGGTAGACGGTCAAGG  Antisense: GGGTAATCATCACATGGTTC | 87 |

**Supplementary Table 1. Oligonucleotides used for qPCR analysis.**

**Supplementary Figure S1.** Anti-biofilm activities of Mannich bases against *C. albicans*

**Supplementary Figure S2.** Inhibitory effect of C1 and C2 on biofilm formation by *C. albicans* strains, (A) MTCC 183, and (B) MTCC 227.


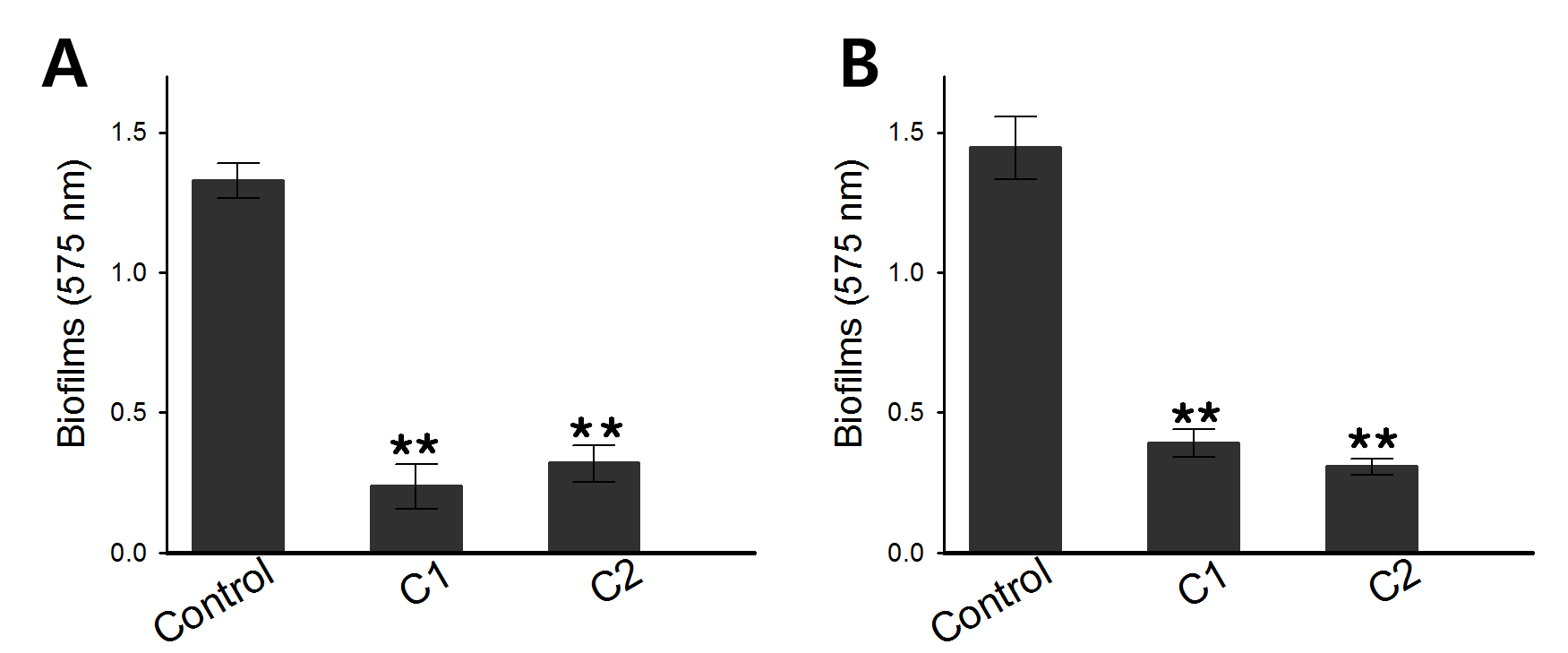


**Supplementary Figure S3.** Glide docking scores of Mannich bases and standard inhibitors with AC
